# How Much Does AMH Really Vary in Normal Women?

**DOI:** 10.1155/2013/959487

**Published:** 2013-11-19

**Authors:** Antonio La Marca, Valentina Grisendi, Georg Griesinger

**Affiliations:** ^1^Mother-Infant Department, Institute of Obstetrics and Gynecology, University of Modena and Reggio Emilia, Policlinico di Modena, Largo del Pozzo, 41100 Modena, Italy; ^2^Department of Reproductive Medicine and Gynecological Endocrinology, University Clinic of Schleswig-Holstein, 23538 Luebeck, Germany

## Abstract

Anti-Mullerian Hormone (AMH) is an ovarian hormone expressed in growing follicles that have undergone recruitment from the primordial follicle pool but have not yet been selected for dominance. It is considered an accurate marker of ovarian reserve, able to reflect the size of the ovarian follicular pool of a woman of reproductive age. In comparison to other hormonal biomarkers such as serum FSH, low intra- and intermenstrual cycle variability have been proposed for AMH. This review summarizes the knowledge regarding within-subject variability, with particular attention on AMH intracycle variability. Moreover the impact of ethnicity, body mass index, and smoking behaviour on AMH interindividual variability will be reviewed. Finally changes in AMH serum levels in two conditions of ovarian quiescence, namely contraceptives use and pregnancy, will be discussed. The present review aims at guiding researchers and clinicians in interpreting AMH values and fluctuations in various research and clinical scenarios.

## 1. Introduction 

Anti-Müllerian Hormone (AMH) is secreted into the circulation by small growing follicles in the ovary, until they have reached the size at which they may be selected for dominance (6–8 mm) [1]. Since the cohort of small growing follicles is in equilibrium with the total number primordial follicles, serum AMH levels reflect the ovarian follicular pool [2]. AMH is therefore considered an accurate marker of ovarian reserve [3, 4]. Moreover AMH levels vary less across different menstrual cycles as well as within one menstrual cycle as compared to other biomarkers of ovarian activity, such as FSH, which has a number of obvious clinical advantages [4–7]. Indeed, according to different studies, the measurement of AMH on a random basis throughout the menstrual cycle is associated with a very good accuracy when predicting ovarian response [8–10].

However while first studies reported a very low variability throughout the menstrual cycle [11–14], a number of more recent studies [15–17] indicate a reduction of circulating AMH in the luteal phase, hence raising the question if AMH should better be measured on a fixed day of the menstrual cycles to foster standardization and to allow better cross comparison between individual assessments.

In this review, we shed light on the partly controversial issue of AMH variability, with particular attention on AMH intracycle variability, that has been recently widely debated. Moreover we evaluate the impact of ethnicity, BMI, and smoking behaviour on AMH interindividual variability. Finally we discussed changes in AMH serum levels in two conditions of ovarian suppression, namely contraceptives use and pregnancy.

## 2. AMH Interindividual Variability

**Figure 1:**
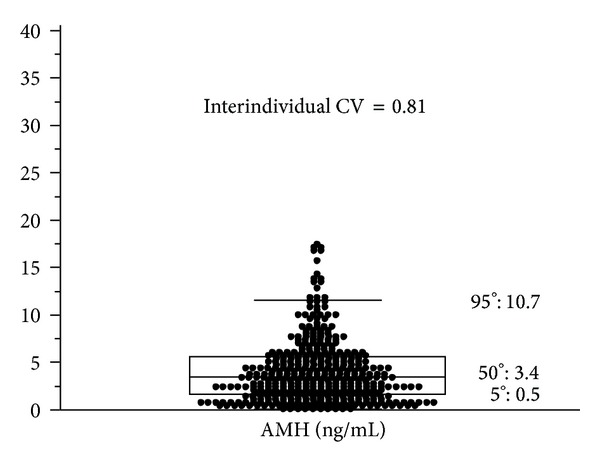
The interindividual variability of AMH refers to variations in AMH levels between different women. The coefficient of variability of AMH in a sample of 416 women aged 18–50 is 0.81. Generally, high interindividual variability is a good characteristic for a hormone when used as discriminatory biomarker in clinical setting (personal data).

When talking about hormonal stability, two different types of variability should be considered: the interindividual and the intraindividual variability. The interindividual variability of AMH refers to variations in AMH levels between different subjects and is first of all secondary to a very high variability in the number of growing follicles within groups of women of similar age [18–20]. The high interindividual variability in AMH is not surprising, given the wide variability of ovarian reserve in women. Generally, high interindividual variability is a good characteristic for a hormone when used as discriminatory biomarker in a clinical setting (Figure 1). Indeed the high interindividual variability of AMH makes it an ideal candidate biomarker with which to discriminate patients for diagnostic, prognostic, and other clinical purposes.

## 3. Effect of Ethnicity, BMI and Smoking on AMH Levels

**Figure 2:**
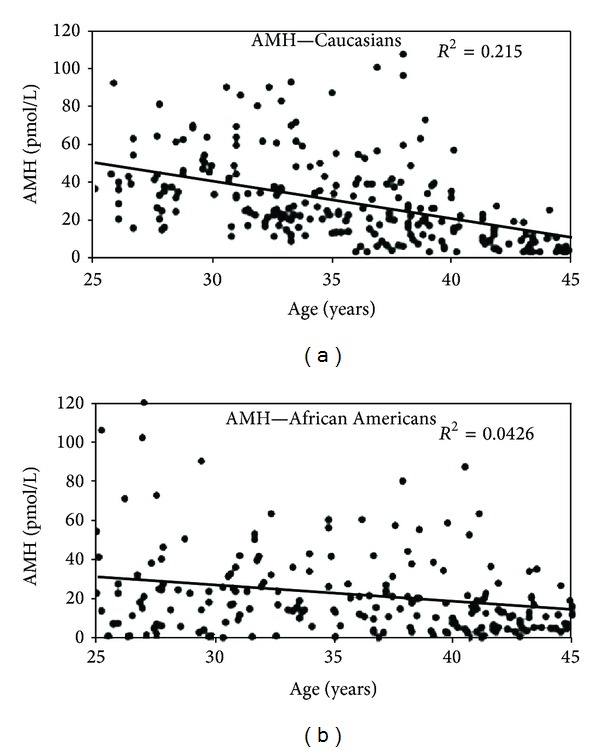
Measurements of AMH versus age in Caucasian (*N* = 232) and African-American women (*N* = 200). Total serum concentrations of AMH versus age indicate that AMH decreases with age but is highly variable between women and is more variable among African-American women. The corresponding correlation coefficients (*R* ^2^) and linear equations are shown. Please note how many African-American women had almost undetectable AMH levels although they were eumenorrhoic (reproduced with permission from Shuh-Huerta et al. [22]).

In uni- and multi-variate analyses, black [21, 22] and Hispanic [21] women exhibit serum AMH levels 25% lower than those found in Caucasian women of similar age. Furthermore, an unexpectedly high number of black women has undetectable AMH levels despite relatively young age and regular menstrual cycles, hence indicating a potential discrepancy between actual ovarian reserve and what is indicated by AMH measurement in this population (Figure 2). More research on the underlying biological phenomena and consequences of this finding is clearly urgently needed. However, this finding indicates that care should be taken when using AMH reference values across different ethnicities.

Some papers, even if limited to small numbers of patients, indicated a negative relationship between BMI and serum AMH levels [23, 24]. However conflicting results have been reported by others [18, 25–28]. In a recent large study performed in a healthy general female population, AMH was negatively related to BMI, but the relationship was age dependent [27]. In other words, in women, AMH levels decreased and BMI increased with age; hence, the relationship between AMH and BMI was only secondary to the stronger relationship of the two variables with age.

**Figure 3:**
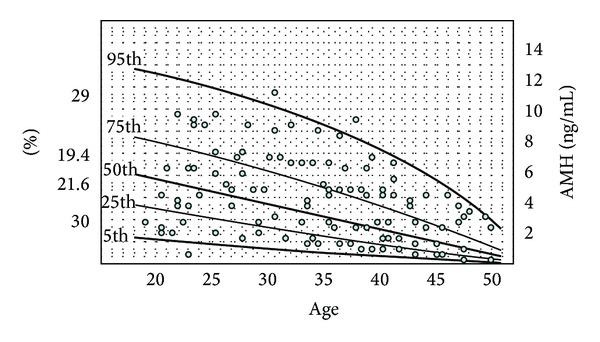
Indifferent distribution of serum AMH levels of smokers (*N* = 99) over quartiles of AMH distribution in the general female population (*n* = 416) (reproduced with permission from La Marca et al. [27]).

There is clear evidence that smoking may directly accelerate ovarian follicular depletion, thereby reducing the age at menopause [29, 30]. Moreover, smoking has been shown to alter metabolic path for several hormones including estradiol. However contradictory results have been reported on the relationship between smoking and AMH, with some authors [31–33] reporting reduced AMH levels in smokers versus nonsmokers and others [18, 27, 34–36] reporting similar values in both groups of women. In a more recent study [27], AMH levels of 416 healthy women, including 99 smokers and 317 nonsmokers, were analyzed. As shown in Figure 3, at any age, the distribution of smokers was uniform in all quartiles of AMH distribution (Figure 3). In other words, in reference to a given age, a similar number of smoking women had high or low AMH levels, respectively. Accordingly, the debate on the impact of smoking on the follicular pool and the circulating AMH levels has not yet been settled. In conclusion, according to the published studies, it seems that the variability in ovarian reserve and secondly ethnicity may largely explain the high degree of interindividual variability in AMH levels. 

## 4. AMH Intraindividual Variability: Long Term, Short Term, and Ultrashort Term

The intraindividual variability is indicative of variations in AMH levels in one single subject and may be secondary to true biological variations in levels of circulating AMH in women.

We propose to distinguish among a long-term variability, a short-term variability and an ultra-short-term variability. The first refers to variations in AMH levels that occur year after year and are indicative of the decline in the ovarian reserve of a single woman. The second depends on the monthly physiologic variability in ovarian function; hence, the short-term variability may refer to intermenstrual cycle variability. The ultra-short-term variability indicates the day-by-day variability and refers to intramenstrual cycle variability.

In a recent prospective longitudinal study, serum AMH levels have been measured in healthy young prepubertal girls (6 to 13 years of age) every 6 months for 3 years and the mean intraindividual coefficient of variation (CV) for AMH was reported as 22%. This indicates that circulating AMH shows—on average—only minor fluctuations within a limited time span; thus, a random AMH measurement is likely to be representative indeed for a given girl [37]. The long, term variability in adult women has been mainly studied in cross-sectional studies, with some of them including as many as 10–15 thousand patients [18, 27, 38–41]. Overall, the studies are in good agreement that AMH declines with advancing age with a pattern that recalls the exponential decay of the primordial follicular pool [2, 42], which is best described by a quadratic equation [38].

**Figure 4:**
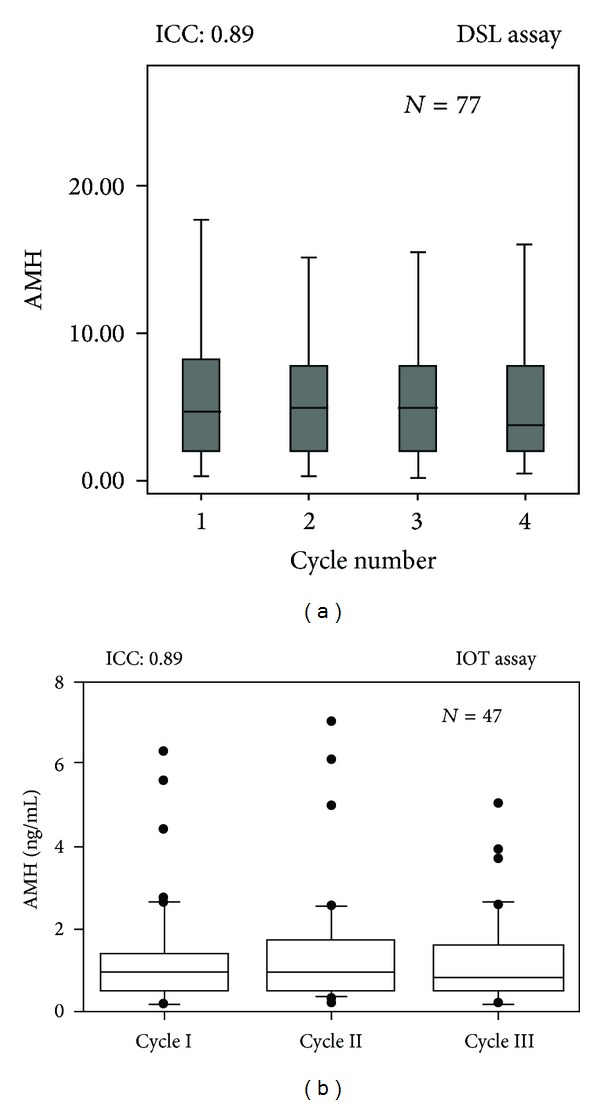
AMH intermenstrual cycle variability throughout several consecutive menstrual cycles. The reported intraclass coefficient (ICC) was 0.89 (reproduced with permission from van Sowers et al. [16] (a) and Fanchin et al. [43] (b)).

 The intermenstrual cycle variability has been analyzed in two well conducted prospective studies [14, 43]. Both studies calculated a similar intraclass coefficient (ICC) which was 0.89. The ICC is the ratio of the interindividual variability over the total variability. Hence the higher the ICC, the lower the intraindividual variability. Both studies concluded that 89% of the variation in AMH was due to between-subject variation, while only 11% of variability was secondary to individual fluctuation in AMH levels (Figure 4). Furthermore, a recent prospective study reported a correlation of 0.88 between AMH measurements performed on cycle day 2 or 3 in two subsequent cycles in women with regular spontaneous cycles [44]. AMH showed the highest between-cycle-correlation within an array of hormones assessed, including testosterone, FSH, E2, inhibin B, and LH.

**Figure 5:**
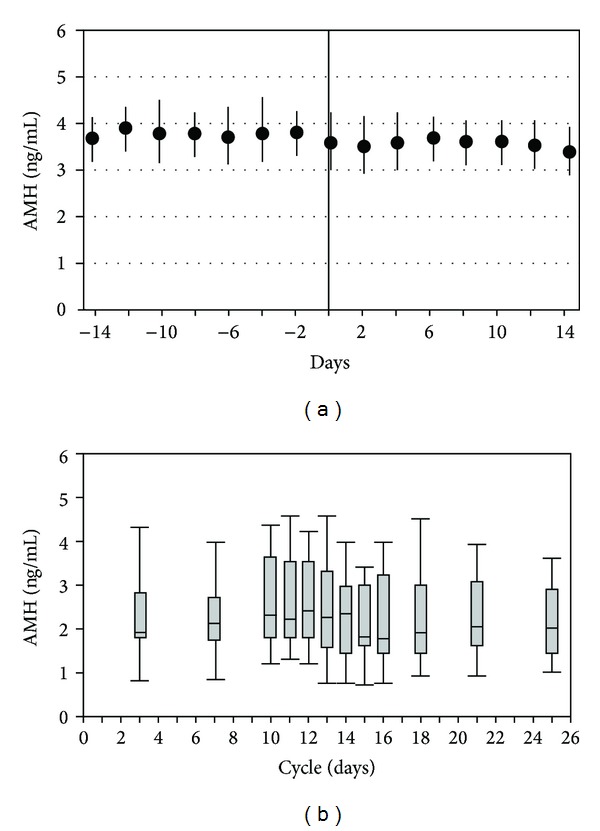
The AMH variability throughout the menstrual cycle. AMH appears to be stable (reproduced with permission from (a) La Marca et al. [12]; (b) Tsepelidis et al. [13]).

A highly debated issue relates to whether AMH significantly varies or not throughout the menstrual cycle. Several studies have suggested that serum AMH levels fluctuate little during the menstrual cycle, as would be expected from the evidence that AMH is not secreted by the dominant follicle or corpus luteum [11–14] (Figure 5). AMH is unique among the known hormones produced by antral follicles, because its secretion seems to be only marginally influenced by gonadotropins and it is dramatically reduced as follicles reach the full gonadotropin sensitivity. As a consequence, AMH levels during the follicular phase do not reflect the activity of the developing large dominant follicle of the month, and conversely on any time point of the menstrual cycle AMH levels provide information on the number of small antral follicle present in the ovary which are available for cyclic follicular recruitment.

To study the intraindividual variability of AMH, Van Disseldorp et al. [14] calculated the intraindividual CV in a reanalyses of a previously published paper [11]. The authors reported that the intraindividual variability of AMH was only 13% and, most importantly, when dividing patients into quintiles according to basal AMH levels, the intraindividual fluctuations were shown to fall in the same quintile in 72% of the cases and to cross two quintiles in only 1% of the cases [14].

**Figure 6:**
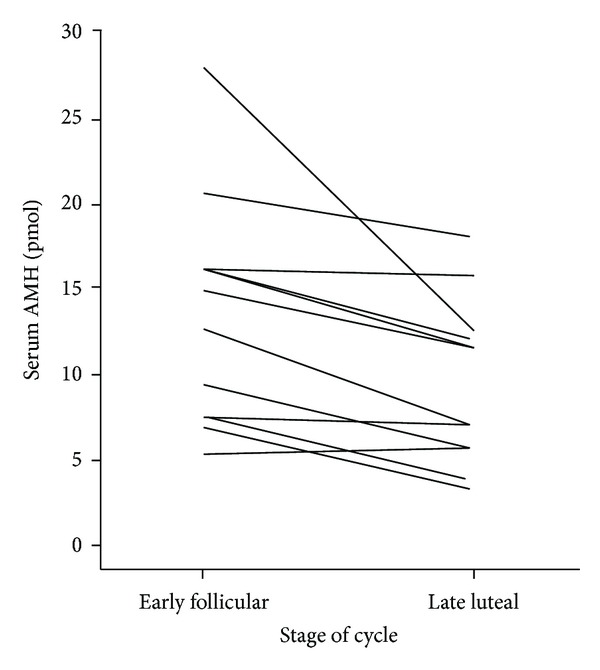
Changes in concentration of AMH for 12 women between the early follicular phase and late luteal phase of the cycle (reproduced with permission from Hadlow et al. [17]).

In contrast, some authors have noted significant fluctuations within one menstrual cycle [15–17]. A very recent study found serum AMH levels significantly lower in the luteal than follicular phase with a variation pattern similar to pituitary FSH, and the intraindividual variance of AMH was as high as 80% [17]. However the study was based on a very small group of subjects (*n* = 12), and some of them had as few as five blood samples throughout an entire menstrual cycle. Moreover when analyzing values for single patients, the proposed decline of AMH in the luteal phase was not evident in 25% of patients (Figure 6), hence raising the questions if the observed reduction of AMH in the luteal phase might be simply casual instead due to a biological reason indeed.

In another prospective study including 20 women, serum AMH levels were shown to fluctuate throughout the menstrual cycle [16]. In this case, the observed fluctuations were absolutely random throughout the cycle and not associated to typical gonadotropin or ovarian steroid patterns. Moreover, the amplitude was proportional to basal AMH levels: women with low AMH levels exhibited only minor fluctuations, whereas women with high basal AMH levels showed relatively higher fluctuations. The author speculated to categorize AMH pattern in “the ageing ovary pattern” and “the younger ovary pattern” [16]. In spite of the good quality of the study, where blood samples were collected from each woman daily along a whole menstrual cycle, some criticisms have been put on Roberts paper [45] for the lack of the calculation of the intraindividual coefficient of variation, which is considered the optimal analysis for hormonal variability. However, at bottom line, Sowers et al.'s study [16] indicates in a clear and convincing way that serum AMH levels vary throughout the menstrual cycle, that fluctuations may be relevant in those women with high basal levels, and most importantly that fluctuations are randomly distributed during the cycle. The random and noncyclic fluctuations in AMH indicate that measuring the hormone on a fixed day of the menstrual cycle would not yield any advantage of a random assessment, for example, on any day of the menstrual cycle.

In order to verify the effect of female age on the degree of AMH fluctuations, a recent study re-evaluated for the third time the data previously described by Hehenkamp et al. [11] and Van Disseldorp et al. [14]. In a group of 44 women between 25 and 46 years of age, the absolute intraindividual variation of AMH (deltaAMH), that is, the difference between maximum and minimum serum level throughout one cycle, was found to be significantly and negatively associated with age. In other words, younger women had significantly larger fluctuations in AMH levels than older women [28]. It may be concluded that in patients with low ovarian reserve (usually aged women), AMH fluctuations have little clinical relevance, while in young patients with usually high ovarian reserve, fluctuations of AMH might indeed impact on the discriminatory capability of diagnostic and predictive tests, respectively [28].

 The observed variability in AMH levels may have a limiting effect on the main current application of AMH as a predictive test in IVF practice. AMH is widely used to predict the ovarian response and to individualize the treatment according to this prediction [5, 9, 46, 47]. If AMH values cross the cut-off values proposed for the ovarian response categories because of true biological variability in AMH, this might lead to misclassification and erroneous treatment of patients. Hence the impact of the documented AMH variability needs to be tested in a clinical setting on a typical target population undergoing a clinically relevant predictive testing scenario.

As reported in detail in several reviews and metanalysis [5, 48–50], AMH is the best hormonal marker for the prediction of ovarian response in IVF. When using a random AMH measurement in order to prospectively predict ovarian response to exogenous FSH, correct categorization of 75% of patients in the three categories poor, normal, or hyper-responder can potentially be obtained [9]. Accordingly, although not as stable as thought before, AMH still remains the most “reliable” ovarian hormone and the best hormonal predictor of ovarian response to stimulation in IVF, with the important advantage of being randomly measurable.

## 5. AMH Serum Levels in Ovarian Quiescence Induced by OC Intake and Pregnancy

Since AMH production by antral follicles has been considered to be largely gonadotropin independent, a logical consequence is that pituitary suppression, as occurring with oral contraceptives (OC) or physiologically during pregnancy, should not be associated with relevant changes in serum levels.

A number of studies have been published on the effect of OC on AMH levels [24, 44, 51–57] and most of the available evaluations are not prospective. The very few prospective studies unfortunately limited the analysis to a few observational months (1 to 4 months) of OC treatment and are thus potentially and insufficiently informative. OC use has been reported either to insignificantly influence AMH concentration [44, 51, 52, 54, 55] or to reduce it significantly [53, 56–58]. 

A large cross-sectional study compared 180 and 76 twenty-year old OC users and nonusers, respectively, and found that long-term OC use was associated with a significant mean reduction in AMH levels by 13% [53]. Recently a cohort study based on 863 healthy women (228 OC users and 504 nonusers) reported that AMH serum levels were 29.8% lower in OC users than those in nonusers. The reduction in AMH was more pronounced with increasing duration of hormonal contraception. However no dose-response relation was found between the dose of ethinyl estradiol and the impact on serum AMH concentration [58].

In a well-conducted prospective study, AMH levels during OC pill intake in long-term OC users (*n* = 25) and 2 months after stopping the OC were assessed. Interestingly, a mean increase in AMH levels by 30% after cessation of the OC was found (from a mean of 2.0 ng/mL during OC to a mean of 2.6 ng/mL two months after the last pill, *P* = 0.001) [56]. 

**Figure 7:**
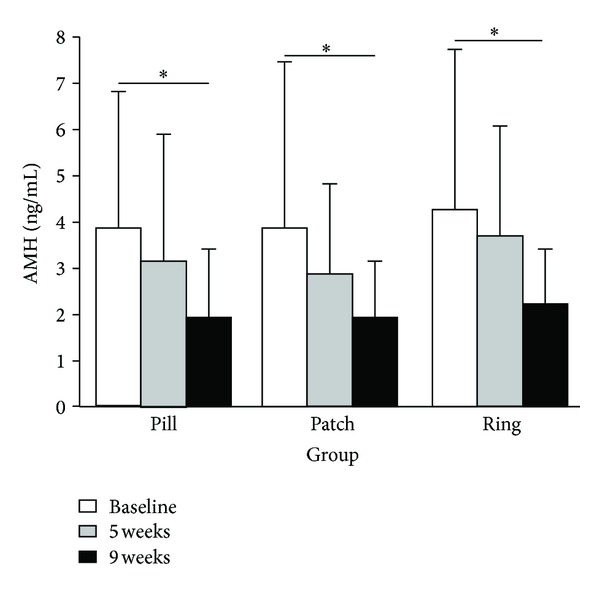
Serum AMH at baseline and after 5 and 9 weeks of administration of contraceptives (reproduced with permission from Kallio et al. [57]).

Finally, a small randomized trial recently confirmed largely these findings [57]. The study population consisted of 42 healthy women randomized to use for 9 weeks an OC in the form of either an oral pill (*n* = 13), a transdermal patch (*n* = 15), or a vaginal ring (*n* = 14). After 9 weeks of contraceptive use, serum AMH levels decreased significantly by almost 50% as compared to baseline in all treatment groups (Figure 7) [57].

This evidence is indicative of a suppressive effect of hormonal contraception on circulating AMH levels, at least when considering long-term use. Thus, serum AMH concentration may not retain its accuracy as predictors of the ovarian reserve in women using hormonal contraceptives for long time.

Pregnancy is a physiological condition associated with ovarian suppression because of suppressed endogenous gonadotropin release. According to the concept that AMH reflects the continuous FSH-independent noncyclic growth of small follicles in the ovary, it would be expected to find nonrelevant alteration in its levels during pregnancy. Indeed, an early small cross-sectional study reported unmodified AMH levels throughout pregnancy [59]. Subsequent studies reported contradictory results, with some confirming this finding [60, 61], while others describing a decrease in AMH levels during pregnancy [62, 63]. It has also been reported that the decline in AMH during pregnancy is evident when using the Beckman Coulter but not the DSL assay [63]. However, in the only longitudinal study available (*n* = 60), authors found a significant decrease in AMH levels in the 2nd and 3rd trimesters compared to the 1st trimester and the mean reduction at the end of pregnancy was of about 50% [64]. This study indicated that during pregnancy, there is a relative ovarian quiescence and reduced follicular maturation with a consequent decrease in the population of follicles secreting AMH. At the same time, at least part of the observed reduction in AMH levels during pregnancy could also be explained by the pregnancy-associated hemodilution and increased plasma-protein binding. 

## 6. Conclusions

In conclusion, on top of the age related decline in AMH, significant fluctuations have been reported for a number of conditions and this has to be taken into account when interpreting values in clinical practice. Fluctuations in the menstrual cycle appear to be random and minor. This suggests that in clinical practice, AMH can be measured independently of the cycle phase. Prolonged ovarian suppression as induced by physiological or pharmacological interventions may reduce AMH levels, since the long and profound pituitary gonadotropin suppression is associated with a reduced number of antral follicles. The exact role of patients' characteristics, as ethnicity, and some habits, as smoking, on intra- and interindividual variability of AMH need to be investigated further.
